# The C-type lectin DCIR contributes to the immune response and pathogenesis of colorectal cancer

**DOI:** 10.1038/s41598-024-57941-y

**Published:** 2024-03-26

**Authors:** Giulia Trimaglio, Tamara Sneperger, Benjamin B. A. Raymond, Nelly Gilles, Emmanuelle Näser, Marie Locard-Paulet, Marieke E. Ijsselsteijn, Thomas P. Brouwer, Romain Ecalard, Jessica Roelands, Naoki Matsumoto, André Colom, Myriam Habch, Noel F. C. C. de Miranda, Nathalie Vergnolle, Christel Devaud, Olivier Neyrolles, Yoann Rombouts

**Affiliations:** 1 Institut de Pharmacologie et de Biologie Structurale, IPBS, Université de Toulouse, CNRS, UPS, Toulouse, France; 2 Department of Pathology, Leiden University Medical Center, Leiden, The Netherlands; 3 INSERM US006 ANEXPLO/CREFRE, Purpan Hospital, Toulouse, France; 4 Department of Integrated Biosciences, Graduate School of Frontier Sciences, The University of Tokyo, Kashiwa, Chiba Japan; 5 Institut de Recherche en Santé Digestive, IRSD, Université de Toulouse, INSERM, INRAe, ENVT, UPS, Toulouse, France

**Keywords:** Dendritic cell immunoreceptor, C-type lectin, Colorectal cancer, Tumor microenvironment, Immune response, Tumour immunology, Cancer models, Glycobiology, Colorectal cancer

## Abstract

Development and progression of malignancies are accompanied and influenced by alterations in the surrounding immune microenvironment. Understanding the cellular and molecular interactions between immune cells and cancer cells has not only provided important fundamental insights into the disease, but has also led to the development of new immunotherapies. The C-type lectin Dendritic Cell ImmunoReceptor (DCIR) is primarily expressed by myeloid cells and is an important regulator of immune homeostasis, as demonstrated in various autoimmune, infectious and inflammatory contexts. Yet, the impact of DCIR on cancer development remains largely unknown. Analysis of available transcriptomic data of colorectal cancer (CRC) patients revealed that high DCIR gene expression is associated with improved patients’ survival, immunologically "hot" tumors and high immunologic constant of rejection, thus arguing for a protective and immunoregulatory role of DCIR in CRC. In line with these correlative data, we found that deficiency of DCIR1, the murine homologue of human DCIR, leads to the development of significantly larger tumors in an orthotopic murine model of CRC. This phenotype is accompanied by an altered phenotype of tumor-associated macrophages (TAMs) and a reduction in the percentage of activated effector CD4^+^ and CD8^+^ T cells in CRC tumors of DCIR1*-*deficient mice. Overall, our results show that DCIR promotes antitumor immunity in CRC, making it an attractive target for the future development of immunotherapies to fight the second deadliest cancer in the world.

## Introduction

Cancers develop in complex microenvironments where neoplastic cells are in continuous cross-talk with the cellular components of both innate and adaptive immunity. Myeloid cells, including monocytes, macrophages, dendritic cells and neutrophils, are the most abundant immune cells within the tumor microenvironment. These cells play diverse and even opposing roles during tumor development^1^. For instance, tumor-infiltrating monocytes can differentiate into phenotypically and functionally distinct tumor-associated macrophages (TAMs) that can exert either antitumor or tumor-promoting functions^1^. Therapies targeting tumor-infiltrating myeloid cells have been recently developed and have shown very promising efficacy, both in pre-clinical and clinical settings^2^. One of these strategies aims to deplete pro-tumorigenic TAMs or reprogram them into antitumor macrophages by using drugs or antibodies directed against cell surface receptors, such as the macrophage colony-stimulating factor receptor and the tumor necrosis factor receptor superfamily member 5^2–4^.

Myeloid cells express a plethora of pattern recognition receptors, including C-type lectin receptors (CLRs), which contribute to the activation of innate immune response and the orchestration of adaptive immunity. CLRs have been traditionally studied for their capacity to bind to various microbial ligands, particularly glycoconjugates, and for their crucial role in antimicrobial immunity^5^. Yet, some CLRs also recognize endogenous ligands such as damage-associated molecular patterns and tumor-associated carbohydrate antigens exposed by cancer cells^6^. Among CLRs, the mannose receptor, Mincle, Dectin-1 and Dectin-2 have been identified as important positive or negative regulators of tumor progression, making these receptors attractive targets for the development of new cancer immunotherapies^6–8^. For instance, specific targeting of the mannose receptor has been shown to selectively reduce immunosuppressive TAMs, increase cancer cell phagocytosis by reprogrammed TAMs, and improve innate and adaptive antitumor immune responses^7^. Likewise, administration of β-glucan, a ligand of Dectin-1, has proven to be efficient in modulating the immune response and inhibiting tumor growth both in pre-clinical and clinical studies^8^.

The dendritic cell immunoreceptor (DCIR) is a CLR almost exclusively expressed by myeloid cells^9,10^. DCIR bears an intracellular immunoreceptor tyrosine-based inhibitory motif (ITIM) and as such is considered as a negative regulator of the immune response^11^. Humans possess a single gene (*CLEC4A*) encoding DCIR, while four DCIR homologs, namely DCIR1-4, are present in the mouse genome^12^. The majority of preclinical studies have been carried out on DCIR1, encoded by *Clec4a2*, due to it containing an intracellular ITIM motif and having a similar expression pattern in myeloid cells as its human counterpart DCIR^9–13^. Several in vitro and in vivo studies have highlighted the immunomodulatory role of human DCIR and/or mouse DCIR1 in various autoimmune disorders^14–16^, infectious diseases^17–19^ and inflammatory-related pathologies^20–24^. However, depending on the pathological context, DCIR1 has been shown to either promote or limit the innate and/or adaptive immune response, demonstrating that the role played by this lectin on immune homeostasis is more complex than initially thought.

Here, we sought to examine the involvement of DCIR/DCIR1 in cancer development and progression, which remains largely unexplored^24–29^. By analyzing publicly available cancer transcriptomic data, we found a positive association between high *CLEC4A* expression and prolonged survival in patients with colorectal cancer (CRC). *CLEC4A* expression is also associated with immune cell infiltration and high immunologic constant of tumor rejection, suggesting a protective role for DCIR during CRC development. To validate these findings, we used an orthotopic syngeneic mouse model of CRC and observed impaired activation of tumor-infiltrating macrophages and T cells along with increased tumor growth in *Clec4a2*^*−/−*^ mice lacking DCIR1 compared to control mice. In summary, our study highlights DCIR as an essential player in CRC immunity, paving the way for further studies to better understand the role of DCIR in tumor development and, in the longer term, for the development of pharmacological agents targeting DCIR to fight certain cancers and beyond.

## Materials and methods

### Human dataset and survival analysis

**Figure 1:**
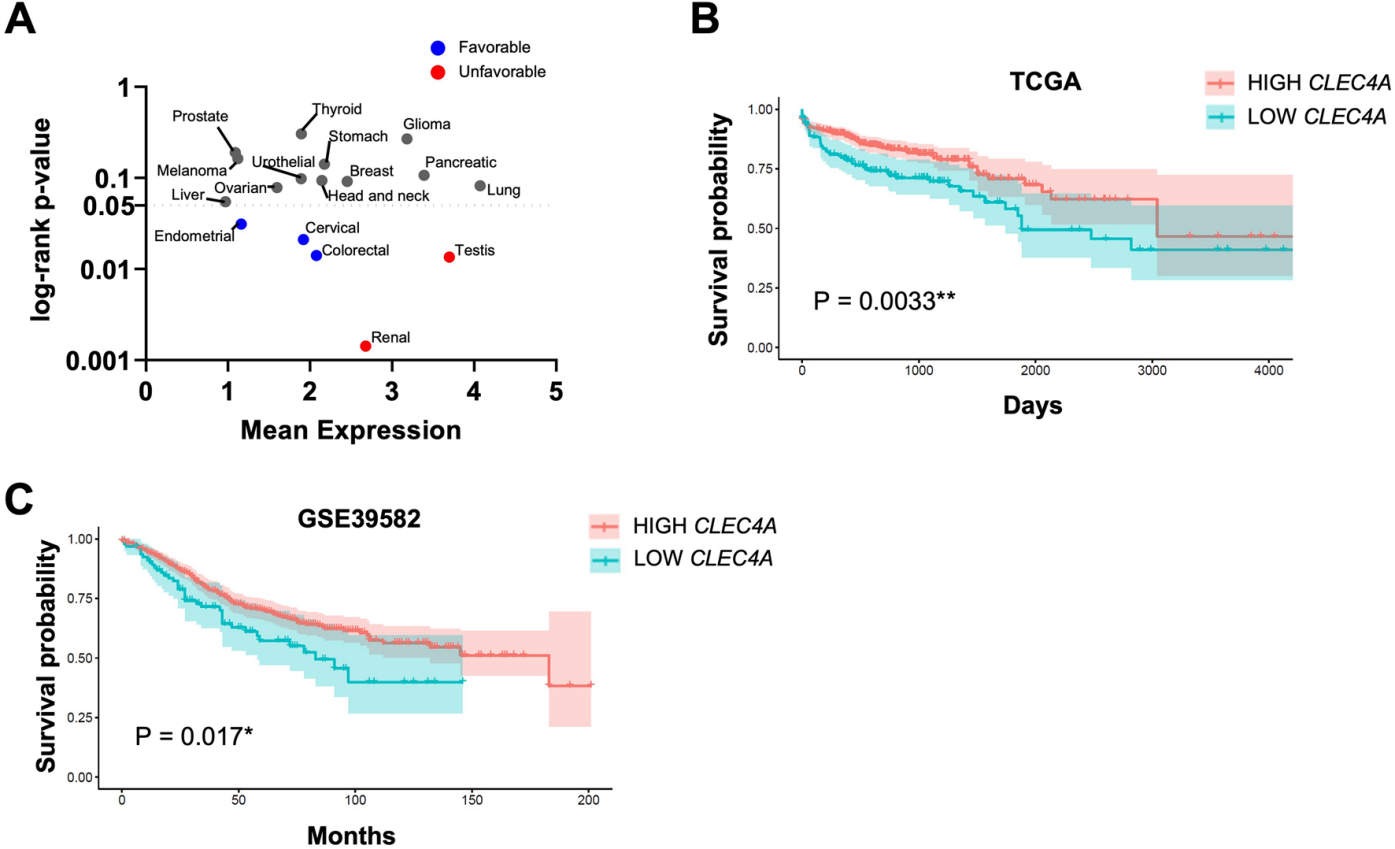
*CLEC4A* expression correlates with an improved CRC patient’s survival. (**A**) *CLEC4A* expression in 17 different types of cancer and its association with cancer patient’s survival^28^. Y-axis represents the mean of *CLEC4A* Fragments Per Kilobase Million (FPKM) in the 694 cancer patients. X-axis (in log scale) represents the log-rank p-value indicating the predictive power of *CLEC4A* expression for cancer patient’s survival. *CLEC4A* expression correlates with an improved (Favorable) or with a worse (Unfavorable) patients survival probability. (**B**,**C**) Overall survival curves from CRC patients exhibiting high and low *CLEC4A* expression in their tumors. Transcriptomic data were obtained from the (**B**) TCGA-COREAD dataset (colon and rectal cohorts combined) (high *CLEC4A*, n = 516, low *CLEC4A*, n = 178)^29^ and from the (**C**) GSE39582 dataset (high *CLEC4A*, n = 486, low *CLEC4A*, n = 93)^30^. A log-rank test was used for the statistical analysis. *P < 0.05, **P < 0.01.

The mean expression levels in Fragments Per Kilobase Million (FPKM) of *CLEC4A* in human cancers (TCGA database; https://portal.gdc.cancer.gov) and the results of *CLEC4A*-centric patient survival analysis (log-rank p-value) presented in Fig. 1A were obtained from Uhlen et al*.*^30^. For the survival analysis presented in Fig. 1B, the expression levels of *CLEC4A* in primary tissue CRC samples were extracted from the Cancer Genome Atlas (TCGA, n = 694 patients) and GSE39582 (n = 579 patients)^31^. TCGA data from colon adenocarcinoma (COAD) and rectum adenocarcinoma (READ) were retrieved using the package TCGAbiolinks on the 25/05/2020 (transcriptome profiling, HTSeq FPKM-UQ). The mRNA expression data GSE39582 were retrieved using GEOquery (v2.54.1). The *CLEC4A* identifiers in the two data sets were ENSG00000111729 and 221724_s_a (*CLEC4A* probe with the highest signal and lowest CV overall). The survival curves were plotted using the R language (v3.6.3) and Cox proportional hazards model (function coxph and survfit from the survival package v3.1–12). The assessment of the predictive power of *CLEC4A* expression for survival was performed using maximally selected rank statistics (maxstat.test function from the package maxstat v0.7–25, smethod = "LogRank", pmethod = "Lau94"). The resulting cutoffs between “high” and “low” were 21,989.86 and 4.656 for TCGA and GSE39582, respectively. The scripts and data associated to this analysis are freely available under the permissive license Creative Commons Attribution 4.0 International on Zenodo.org with the 10.5281/zenodo.3842597. For generating Fig. 1C–E, transcriptomic data of the TCGA-COAD dataset was downloaded using TCGABiolinks (v2.18) in R (v4.0.3). Gene expression was normalized using the TCGAanalyze_Normalization function to normalize within lanes, to correct for gene-specific effects (including GC-content) and between lanes, to correct for sample-related differences (including sequencing depth) using the EDASeq package. After normalization, samples were extracted to obtain a single primary tumor tissue (TP) sample per patient using the ExtractTissueSpecificSamples function from TCGA Assembler (V.2.0.3). Samples were classified according to Consensus Molecular Subtypes (CMS) by “CMSclassifier” (R, v.1.0) using random forest method^32^. The MANTIS score with threshold 0.4 was used to determine microsatellite status^33,34^. The ICR signature (20 genes) was used to derive the cancer immune phenotype, reflecting the activation of T helper 1 (Th1) cell-related factors (*IFNG*, *TXB21*, *IL12B*, *STAT1*, and *IRF1*), CD8 T cell markers (*CD8B and CD8A*) CXCR3/CCR5 chemokine ligands (*CXCL9*, *CXCL10*, *CCL5*), cytotoxic effector molecules (*GNLY*, *PRF1*, *GZMA*, *GZMB*, and *GZMH*) and compensatory immune regulators (*CD274/PD-L1*, *PDCD1*, *CTLA4*, *FOXP3* and *IDO1*)^35,36^. To derive ICR clusters, consensus clustering based on the 20 ICR genes was performed for each cancer type separately using the ConsensusClusterPlus (V.1.42.0) R package with the following parameters: 5000 repeats, a maximum of six clusters and agglomerative hierarchical clustering with ward criterion (Ward.D2) inner and complete outer linkage. Consensus Tumor Microenvironment cell Estimation (ConsensusTME)^37^ was used to estimate relative abundancies of specific immune cell subsets from bulk transcriptome data. We applied ConsensusTME using ConsensusTME (v.0.0.1.9) using parameters “COAD” to specify cancer type and “ssgsea” as statistical method.

### Human CRC tissues and immunohistochemistry

Patient samples were anonymized and handled according to the medical ethical guidelines described in the Code of Conduct for Proper Secondary Use of Human Tissue of the Dutch Federation of Biomedical Scientific Societies. This study was approved by the Medical Ethical Committee of the Leiden University Medical Center (protocol P15.282), and patients provided written informed consent. A tissue micro array (TMA) of formalin-fixed paraffin embedded (FFPE) CRC tissues was created by the department of pathology of the Leiden University Medical center (Leiden, The Netherlands). Representative tumor regions were selected by the pathologist and the final TMA contained seventy-six 1.5 mm cores, from 22 tumors, which included 12 mismatch repair deficient (MMRd) and 10 mismatch repair proficient (MMRp) cases. 4 µm sections were cut and analyzed by immunohistochemical detection. In detail, the FFPE tissue sections were de-paraffinized using xylene and washed in 100% ethanol. Endogenous peroxidase was blocked by incubation in a 0.3% hydrogen peroxide/methanol (Merck Millipore) solution for 20 min. After washes in 70% and 50% ethanol, heat-induced antigen retrieval was performed using pre-warmed citrate buffer (10 mM, pH 6.0) for 10 min. Following cooling, FFPE tissue sections were first incubated for 30 min at 4 °C with the Superblock solution (Thermo Fisher Scientific) and then overnight at 4 °C with the primary rabbit anti-DCIR antibody (dilution 1:200, Sigma-Aldrich). The following day, slides were washed in Phosphate-Buffered Saline (PBS) and incubated for 1 h at room temperature with BrightVision poly HRP anti-mouse/rabbit IgG (Immunologic, Duiven, The Netherlands). Antibody binding was developed with the DAB^+^ chromogen (DAKO, Agilent Technologies) solution and the slides were counterstained with hematoxylin (Thermo Fisher Scientific). Staining was evaluated and scored using a light microscope. Tissue cores that could not be evaluated due to poor quality or absence of an internal control were excluded.

### Mice

All animal experimental procedures were approved by the regional Ethic Committee of Toulouse Biological research Federation and by the Ministry of Higher Education and Research (APAFlS#5168 and APAFlS#16904). All methods were carried out in accordance with the European directive 2010/63/EU and reported in the manuscript following the recommendations of the ARRIVE guidelines. DCIR1 deficient mice (*Clec4a2*^*−/−*^) were provided by the NIH-sponsored Mutant Mouse Regional Resource Center and were originally generated by the Consortium for Functional Glycomics. The strain was backcrossed for more than 10 generations on a C57BL/6 background. Animals used in this study consisted of WT and *Clec4a2*^*−/−*^ mice originating from common heterozygous (*Clec4a2*^−/+^) mice. Details for genotyping are available on the CFG website. WT and *Clec4a2*^*−/−*^ mice were bred in an EOPS protected area (free of specific pathogens) within the animal facility of the Institute of Pharmacology and Structural Biology and housed for the experiments in an EOPS protected area within the animal facility of the Centre Hospitalier Universitaire de Toulouse-Purpan. In vivo experiments were performed using 6- to 12-week-old *Clec4a2*^*−/−*^ mice and their aged-matched WT controls by qualified personnel and in animal facilities that met all legal requirements in France, thus minimizing animal discomfort and suffering. At the end of each experimental procedure, mice were sacrificed by cervical dislocation under anaesthesia (4% isoflurane, vetflurane Virbac Denmark).

### Tumor cell lines

MC38 cells expressing firefly luciferase (MC38-fLuc^+^) were generated from MC38 parental cell line (Kerafast, RRID:CVCL_B288) as previously described^38,39^ and kindly provided by Dr Myriam Capone and Sonia Netzer (ImmunoConcept, CNRS UMR5164, University of Bordeaux, France). B16F1 and B16F10 (ATCC, RRID:CVCL_0158 and RRID:CVCL_0159 respectively) are low and high aggressive metastatic melanoma cell lines, respectively^40^. Cells were cultured at 37 °C and 5% CO2 in Dulbecco’s Modified Eagle’s Medium high glucose, GlutaMAX supplement, pyruvate (DMEM GlutaMax, Sigma-Aldrich) supplemented with 10% FBS (Sigma-Aldrich) and 1% Penicillin–Streptomycin (Gibco™). After cell thawing, additional 1% L-glutamine (Gibco, Thermo Fisher) was added to the culture medium to ensure better recovery of the cells. Passage of the cells (2 to 6 passages maximum) was performed using Cell Dissociation Solution Non-enzymatic 1x (Sigma-Aldrich) or Trypsin–EDTA 1× (Gibco) when approximately 70% of cell confluence was reached. All cells were regularly tested for mycoplasma contamination and all experiments were performed with mycoplasma-free cells.

### Tumor implantation and monitoring of tumor development

For the generation of CRC tumors, 2 × 10^6^ viable MC38-fLuc^+^ were implanted intra-colon (IC), into the caecum of mice (6- to 9-week old) that were anesthetized beforehand via intra-peritoneal (IP) administration of ketamine/xylazine, as previously described^39^. Monitoring of tumor growth was performed twice a week using the cooled charge-coupled device camera IVIS Spectrum in vivo Imaging System (PerkinElmer) after IP injection of the mice with 150 mg/kg of D-Luciferin (Oz Bioscience). The bioluminescent signal intensity, presented as average radiance (photons/sec/cm^2^/sr), was analyzed using IVIS Living Image 4.5.2 software (PerkinElmer). Subcutaneous tumors were established by injecting 3 × 10^5^ B16F1, 2 × 10^5^ B16F10 or 1 × 10^6^ MC38- fLuc^+^ viable cells in the right flank of mice (6- to 9-week-old). Tumor progression was measured using a caliper and mice were euthanized when tumor size reached the ethically defined limit of 250 mm^2^.

### Tissue processing

Following mouse dissection, IC tumors were collected, weighed and digested for 5–10 min in DMEM GlutaMax with 1 mg/ml Collagenase Type IV, 50 U/ml DNase I Type IV and 100 μg/ml Hyaluronidase type 4 (all from Sigma-Aldrich) and 1% L-glutamine (Gibco) at 37 °C with agitation. Mesenteric tumor-draining lymph nodes (mLNs) were also collected but were not subjected to enzymatic digestion. Digested IC tumors and non-digested mesenteric tumor-draining lymph nodes (mLNs) were then dissociated by filtration through a 70 μM cell strainer (Clearline) using a sterile syringe plunger. The resulting single-cell suspensions were further filtered through a 40 μM cell strainer (Clearline) and pelleted by centrifugation at 450×*g* for 6 min. Erythrocyte lysis with Ammonium-Chloride-Potassium buffer was performed before the cells were finally resuspended in PBS (DPBS, no calcium, no magnesium, Gibco) containing 2% FBS (Sigma-Aldrich) and 2 mM Ethylenediaminetetraacetic acid (EDTA, Sigma-Aldrich) for cell staining and FACS analysis. To analyze colonic myeloid cells from resting mice, the colon was harvested by cutting the distal part between the cecum and the rectum. The colon was cleaned in HBSS containing Antibiotic–Antimycotic (Gibco) at 4 °C to remove fecal matter before being cut longitudinally and then into small 0.5 cm pieces. Subsequently, Colon pieces were incubated with agitation for 20 min at 37 °C in 10 ml of prewarmed media (RPMI 1640 Glutamax; Gibco) supplemented with 10% FBS (Sigma-Aldrich), 20 mM Hepes, Antibiotic–Antimycotic, 5 mM EDTA and 1 mM freshly thawed dithiothreitol. After incubation, the remaining colon pieces were filtered on a 70 μm cell strainer (Clearline) and repeatedly shaken for 20 min at 37 °C in 10 ml of serum free media supplemented with 20 mM Hepes, Antibiotic–Antimycotic and 2 mM EDTA. Again, the remaining colon pieces were filtered through a 70 μm cell strainer and finally incubated for 30 min at 37 °C in 10 ml of serum free media supplemented with 0.25 mg/ml DNase I (Roche) and 0.1 mg/ml Liberase TM (Roche). Digested colon pieces were passed sequentially through a 70 μm and 40 μm cell strainer, and washed with 10 ml of media supplemented with 10% FBS to stop the digestion. The cells within the media were pelleted by centrifugation prior to cell staining and FACS analysis.

### Cell staining with fluorescent antibodies and FACS analysis

Cells were transferred to V-bottom 96 well plates and pelleted by centrifugation at 600 g for 2 min. Following supernatant removal, cells were incubated for 20–30 min at 4 °C in the dark with a mix of anti-CD16/32 antibodies (TruStain FcX™, Biolegend) to block Fc receptors (avoiding non-specific staining), a viability marker (live/dead fixable blue dead cell stain kit, Invitrogen™) and appropriate antibodies against membrane proteins diluted in PBS containing 2% FBS and 2 mM EDTA (FACS buffer). For the detection of intracellular proteins (transcription factors and CTLA-4), cells were shortly washed with FACS buffer, fixed for 30–45 min at room temperature in the dark (following fixation centrifugation steps were all performed at 700*g* for 2 min), and permeabilized in permeabilization buffer (Foxp3/transcription factor staining buffer set, eBioscience) for 15 min at room temperature in the dark. Cells were then incubated with antibodies directed against intracellular proteins diluted in permeabilization buffer for 45 min at room temperature in the dark. The antibodies and dyes used for FACS staining are listed in Supplementary Table S1. The anti-DCIR1 antibody (TKKT-1)^9^ was coupled to Alexa Fluor™ 647 using antibody Labeling Kit (Thermo Fisher Scientific) according to manufacturer’s instructions. Staining was acquired with a Fortessa flow cytometer (BD Biosciences) and analyzed with the FlowJo V10 software. The gating strategy was performed similarly for all analyses (Supplementary Figs. S4, S8, S10): FSC-A *vs.* SSC-A was performed first, in order to gate on cells and avoid debris, followed by FSC-H *vs.* FSC-A (or alternatively FSC-H vs FSC-W) and SSC-H *vs.* SSC-A (or alternatively SSC-H *vs.* SSC-W) to remove doublets. When mentioned, cell surface protein expression is represented as median fluorescent intensity (MFI) or differential median fluorescent intensity ΔMFI. The latter was calculated as the difference between the MFI of the positive population and the negative one for a given marker. When a negative population was not present for a mouse, a median of MFI of the negative population from the other mice was subtracted from the MFI of the positive population of the specific mouse.

### Generation and interferon stimulation of bone marrow-derived macrophages and dendritic cells

Bone marrow-derived macrophages (BMDMs) and dendritic cells (BMDCs) were generated from bone marrow cells isolated from femurs and tibias from the hind legs of WT or *Clec4a2*^*−/−*^ mice (6- to 12-week-old). Cells were grown in DMEM GlutaMAx (Sigma-Aldrich) containing 10% FBS (Sigma-Aldrich) and 1% Penicillin–Streptomycin (Gibco), and supplemented with 20 ng/ml of recombinant murine macrophage colony-stimulating Factor (M-CSF, PeproTech) for BMDM differentiation or 20 ng/ml of recombinant murine granulocyte–macrophage colony-stimulating factor (GM-CSF, PeproTech) for BMDC generation. The medium was changed every 2–3 days and BMDMs and BDMCs were collected on days 7–9 and 10–12 respectively, using Cell Dissociation Solution Non-enzymatic 1× (Sigma-Aldrich) and cell scraping (Falcon® cell scraper Corning). Cells were transferred in 24-well plates (5 × 10^5^/well) and incubated overnight in DMEM/10% FBS/1% Penicillin–Streptomycin before stimulation for 24 h with indicated concentrations of murine IFN-alpha (Biolegend), IFN-beta (Biolegend) or IFN-gamma (Biolegend). Cells were finally detached as described above, stained with antibodies mentioned in supplementary table S1 and finally acquired on a Fortessa flow cytometer (BD Biosciences) followed by analysis with the FlowJo V10 software.

#### Real-Time qPCR

To extract total RNA, frozen tissues were first collected in M-tubes (Miltenyi Biotec) containing an appropriate volume of TRIzol™ (Invitrogen™), according to manufacturer’s instructions, and homogenized using a gentleMACS™ dissociator (RNA_02 program). Homogenates were resuspended in CHCl_3_ (200 µl for 300 µl of Trizol™), the aqueous phase was collected, and total RNA was precipitated by adding the same volume of ethanol. RNA was purified using RNeasy spin columns, according to manufacturer's instructions (RNeasy Mini kit, Qiagen). cDNA was synthetized from 200 ng of total RNA using the M-MLV Reverse Trancriptase kit (Invitrogen™). Real-Time qPCR reactions were performed using an Applied Biosystems® 7500 Real-Time PCR System (Applied Biosystems) after mixing cDNA with gene-targeted primers (Supplementary table S2) and qPCR Mastermix plus SYBR Green (Eurogenetec). The 7500 Software v2.3 was used for data analysis. The amplification of the genes of interest was normalized on β-actin and values were expressed as 2^−ΔCt^.

### Statistical analysis

Where not specified otherwise, statistical analysis was performed with Prism software (GraphPad Prism). Correlations between CLEC4A expression and survival of patients were analyzed by a log-rank test. Other statistical correlations were calculated using either Pearson or Spearman if normally or not normally distributed respectively. Statistical comparison of bioluminescent curves was performed using a mixed-effects model. The normal distribution of the population was evaluated using D’Agostino-Pearson omnibus normality test, Shapiro–Wilk normality test and Kolmogorov–Smirnov test with the Dallal and Wilkinson approximation to Lilliefors' method. Populations were treated as normal when at least two out of the three tests confirmed a normal distribution. Experiments were analyzed using either unpaired t-test (for populations with normal distribution) or a Mann–Whitney test (for populations without normal distribution). Accordingly, results are expressed as mean ± standard deviation (SD) or median with interquartile range. A p-value (P) < 0.05 was considered significant. *P < 0.05, **P < 0.01, ***P < 0.001, ****P < 0.0001.

## Results

### DCIR gene expression is associated with improved survival in CRC patients

In order to evaluate whether the expression of *CLEC4A*, encoding human DCIR, correlates with the prognosis of cancer patients, we first examined the mean expression of *CLEC4A* (in FPKM) *vs.* the results of *CLEC4A*-centric patients’ survival analysis in 17 prevalent human cancer types (in log-rank p-value), as previously determined using the TCGA database^30^. *CLEC4A* is expressed in all major human cancer types, although its expression varies from one type of cancer to another. Moreover, depending on the cancer type, *CLEC4A* appears to be either an unfavorable or a favorable prognostic gene, meaning that a higher *CLEC4A* expression correlated with a shorter or longer patients’ life expectancy, respectively (Fig. 1A). Among the most significant results, high *CLEC4A* expression was associated with poor prognostic in testis and renal cancer. Interestingly, it was associated with longer patient’s survival in colorectal (CRC), endometrial and cervical cancer, with the lowest log-rank p-value found for CRC. Given that DCIR carries a cytoplasmic ITIM motif and is thought to dampen the immune response, we were intrigued by the positive association between a high *CLEC4A* expression and a longer patients’ survival outcome as observed in CRC. We confirmed that *CLEC4A* was a marker of favorable prognostic in CRC patients by reanalyzing the transcriptomic TCGA data from 694 CRC patient samples as well as by using an independent cohort of 579 CRC samples (Fig. 1B,C)^31^. Further analysis of TCGA data reveals a significant decrease in *CLEC4A* expression from stage I to stage IV of colon cancer (Supplementary Fig. S1A). Moreover, *CLEC4A* expression is associated with longer overall survival of patients with stage I, III, and IV colon cancer (HR < 1), although it is only significant in stage III (Supplementary Fig. S1B). An inverse association, although not significant, was found at stage II.

### DCIR gene expression is associated with immunologically “hot” CRC tumors and correlates with immune cell infiltration

**Figure 2:**
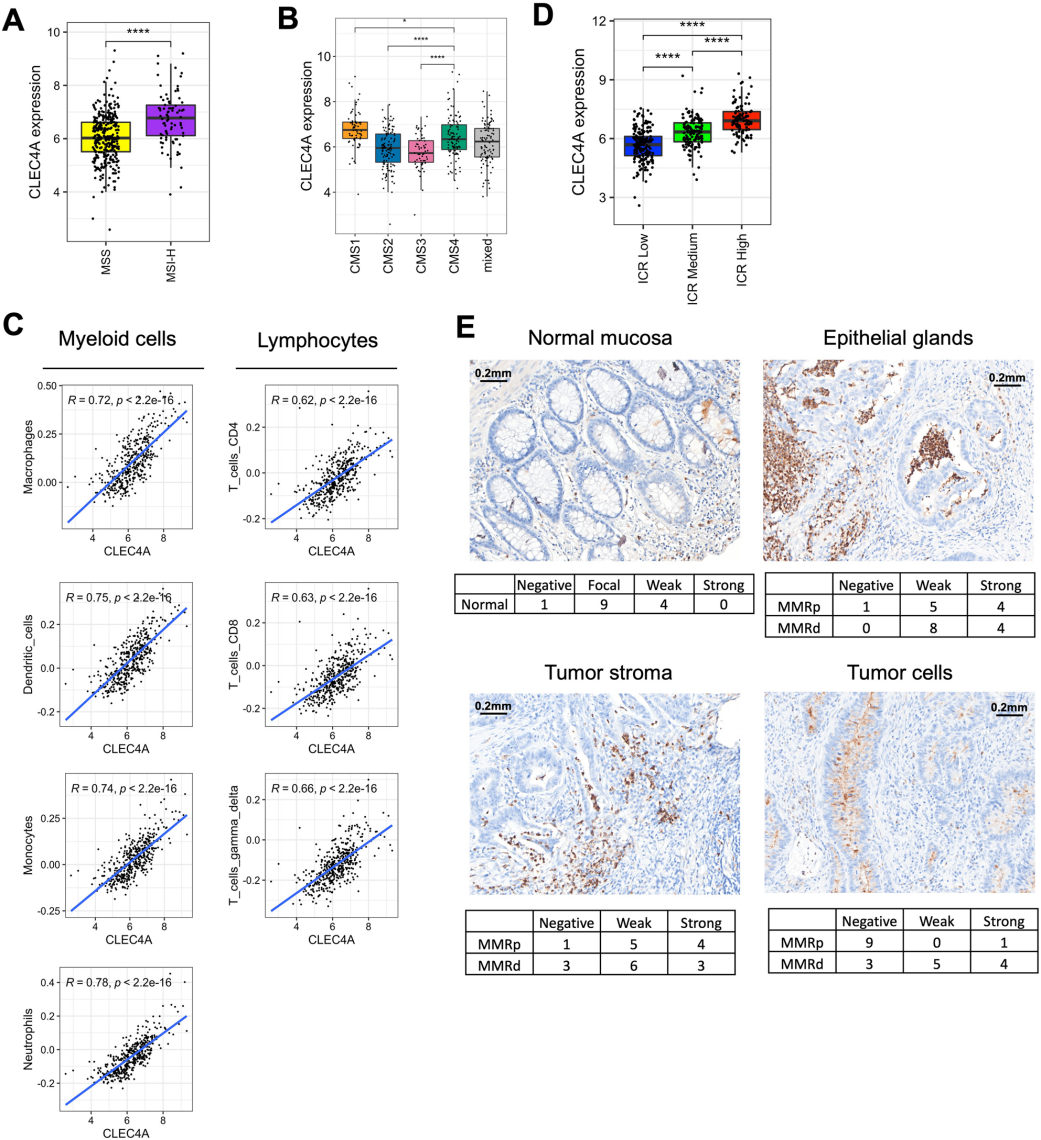
*CLEC4A* expression is associated with immunologically “hot” CRC tumors. (**A**,**B**) *CLEC4A* expression in primary tumor samples of the TCGA-COAD dataset by (**A**) microsatellite instability status (Microsatellite Stable (MSS), n = 331 and high microsatellite instability (MSI-H), n = 82) and by (**B**) consensus molecular subtypes of colorectal cancer (CMS1, n = 64, CMS2, n = 121, CMS3, n = 55, CMS4, n = 103, mixed, n = 96). An unpaired Student’s t-test was used for the statistical analysis. (**C**) Pearson correlation between *CLEC4A* expression in primary tumor samples of the TCGA-COAD dataset (n = 439) and estimated relative abundance of immune cells subsets using deconvolution by ConsensusTME^36^. (**D**) *CLEC4A* expression in TCGA-COAD dataset by Immunologic Constant of Rejection (ICR) clusters (ICR High, n = 107, ICR Medium, n = 146, ICR Low, n = 186). (**E**) Representative immunohistochemical images of DCIR expression detected in the normal mucosa, epithelial glands, tumor stroma and tumor cells of a CRC patients’ cohort (n = 22). Values indicate the numbers of cores presenting negative, focal, weak or strong positive staining for DCIR in MMR-proficient (MMRp) and MMR-deficient (MMRd) CRC. *P < 0.05, ****P < 0.0001.

We next analyzed the association of *CLEC4A* expression with multiple tumor parameters including high microsatellite instability (MSI-H) tumors and consensus molecular subtypes (CMS). *CLEC4A* expression is closely associated with immune cell infiltration and a typical “hot” immune phenotype that is often encountered in high microsatellite instability (MSI-H) tumors and in the consensus molecular subtypes (CMS) CMS1 and CMS4 of CRC (Fig. 2A,B). Using the consensusTME (Tumour microenvironment cell estimation) deconvolution algorithm, we observed a strong correlation between *CLEC4A* expression and abundancies of multiple myeloid cells including macrophages, dendritic cells, monocytes and neutrophils (Fig. 2C), consistent with the fact that all of these cells express DCIR. Interestingly, *CLEC4A* expression also positively correlates with the abundance of distinct T cell subsets (CD4^+^, CD8^+^ or Gamma delta T cells) as well as with a high immunologic constant of rejection (ICR) which reflects an active Th1/cytotoxic immune response (Fig. 2C,D). We then analyzed DCIR presence at the protein level in CRC tumors by performing immunohistochemical analysis on full slides of colorectal tissues from 22 patients (Fig. 2E). DCIR expression was determined focally positive or weak in normal colonic mucosa (evaluable in 14 cases), demonstrating increased expression closer to regions with malignant transformation (dysplasia). In tumor tissues, DCIR expression could, in most cases, be detected inside epithelial glands, often in association with granulocytic infiltrate, in the tumor stroma (82% of cases) on cells with immunological morphology and, occasionally on tumor cells themselves (45% of cases). Interestingly, expression of DCIR on tumor cells was more frequent on mismatch repair (MMR)-deficient cases as compared to MMR-proficient ones (75% vs. 10%). Altogether, these data indicate that *CLEC4A* is expressed both at the mRNA and protein levels in human CRC tumors and suggest that DCIR regulates the anti-tumor immune response in CRC.

### *Clec4a2*^*−/−*^ mice lacking DCIR1 develop larger colon tumors compared to WT animals

**Figure 3:**
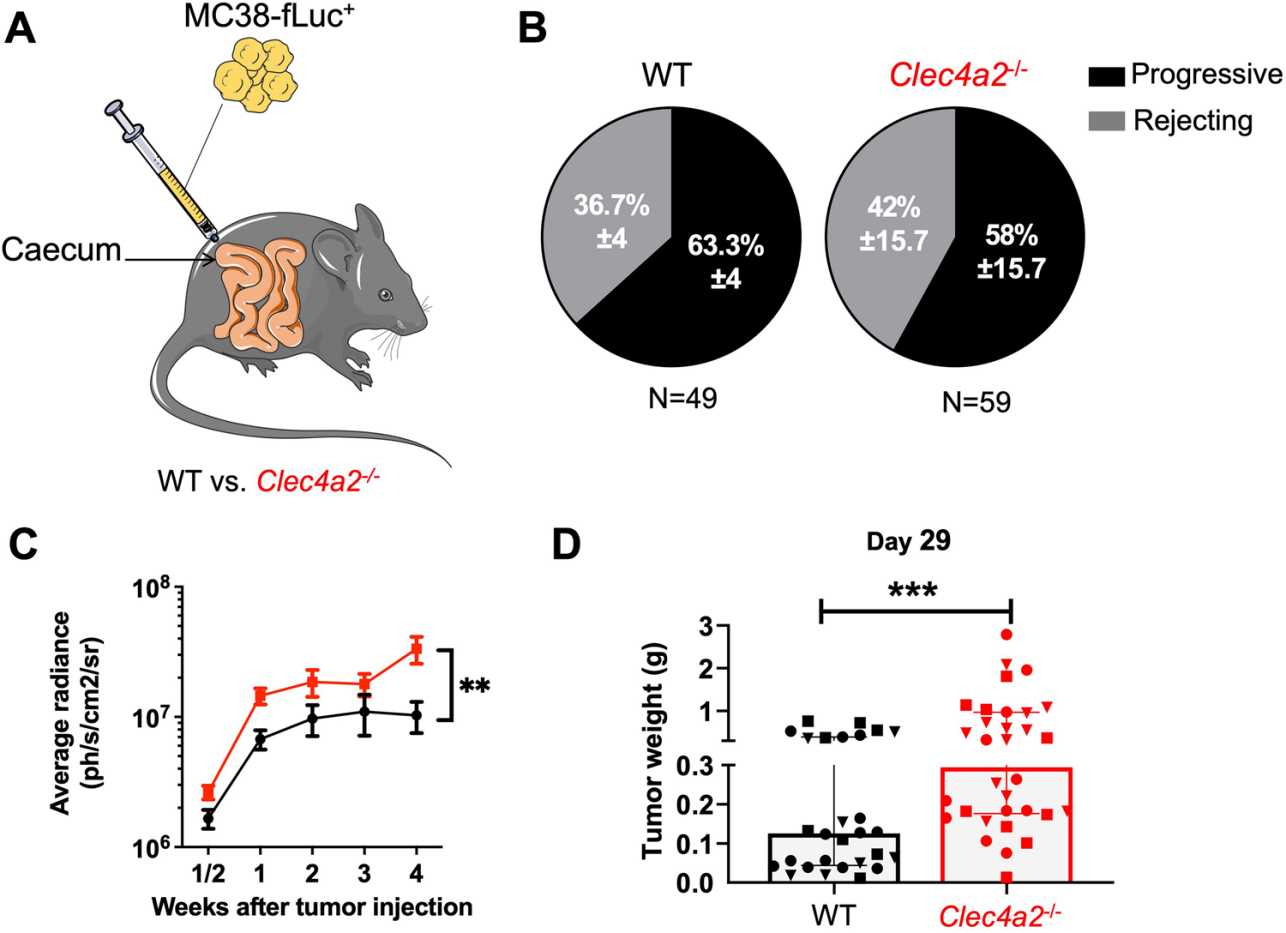
DCIR1 deficient mice developed larger CRC tumors than WT mice. (**A**) Schematic illustrating the orthotopic CRC mouse model used in this study. (**B**) Pie charts showing the mean ± SD of the percentage of progressive and rejecting mice in WT and DCIR1-deficient (*Clec4a2*^*−/−*^) mice at day 29 after IC injection of MC38-fLuc^+^ tumor cells (3 independent experiments pooled). The total number of mice (N) per genotype is indicated below. (**C**) Longitudinal bioluminescence emission (in ph/s/cm^2^/sr; photon per second per square centimeter per steradian) from imaging of WT and *Clec4a2*^*−/−*^ progressive mice following IC injection of MC38-fLuc^+^ cells (3 independent experiments pooled, n = 31 WT and n = 34 *Clec4a2*^*−/−*^ mice). Mean ± SEM in log10 scale is represented. (**D**) Quantification of tumor weight in grams (g) from WT and *Clec4a2*^*−/−*^ progressive mice on day 29 following IC injection of MC38-fLuc^+^ cells (3 independent experiments pooled, n = 28 WT and n = 32 *Clec4a2*^*−/−*^ mice). Each symbol corresponds to a single mouse and the different symbols (*i.e.,* dots, squares, triangles) are used to indicate independent experiments. **P < 0.01, ***P < 0.001.

To better understand the impact of DCIR on CRC pathogenesis, we used an orthotopic syngeneic CRC mouse model that we previously developed and characterized^39^. This model involves the implantation of a MC38 CRC cell line, a model for hypermutated/MSI CRC^41^, that expresses firefly luciferase (MC38-fLuc^+^), (Fig. 3A) into the caecum (IC) of mice. By monitoring tumor growth over time using a bioluminescence camera, we observed that, despite initial tumor growth in all mice, some animals subsequently rejected the cancer cells (rejecting mice) while others developed progressive CRC (progressive mice) as previously reported^39^. However, we found no impact of DCIR1 deficiency on the proportion of rejecting/progressing mice (Fig. 3B). To access the impact of DCIR1 deficiency on CRC development, we compared the longitudinal bioluminescence intensity in WT and *Clec4a2*^*−/−*^ mice with progressive CRC. We observed higher bioluminescence intensity in *Clec4a2*^*−/−*^ mice compared with WT animals, particularly at week 4 after tumor implantation, suggesting increased tumor development in the absence of DCIR1 (Fig. 3C). Concordantly, when mice were sacrificed at day 29 after tumor cell injection, we found that *Clec4a2*^*−/−*^ mice developed significantly larger tumors than WT animals (Fig. 3D).

Given that human melanoma tumor samples have one of the lowest mean expressions of *CLEC4A* and no significant association with survival in melanoma patients (Fig. 1A), we sought to examine the impact of DCIR1 in the development of melanoma in mice. To this end, we subcutaneously injected the less aggressive B16F1 or the more aggressive B16F10 melanoma tumor cells, into WT and *Clec4a2*^*−/−*^ mice and measured tumor volume over time. Regardless of the tumor cell line, we observed a similar rate of tumor growth in both mouse lines (Supplementary Fig. S2A,B). Likewise, after subcutaneous injection of the MC38-fLuc^+^ cells, we found no difference in tumor growth between WT and *Clec4a2*^*−/−*^ animals (Supplementary Fig. S2C). Overall, these results show that, consistent with the human TCGA transcriptomic data analysis, the absence of DCIR1 promotes CRC tumor progression in mice while it has no impact on subcutaneous tumor development.

### DCIR1 deficiency alters the phenotype of myeloid cells infiltrating CRC tumors in mice

**Figure 4:**
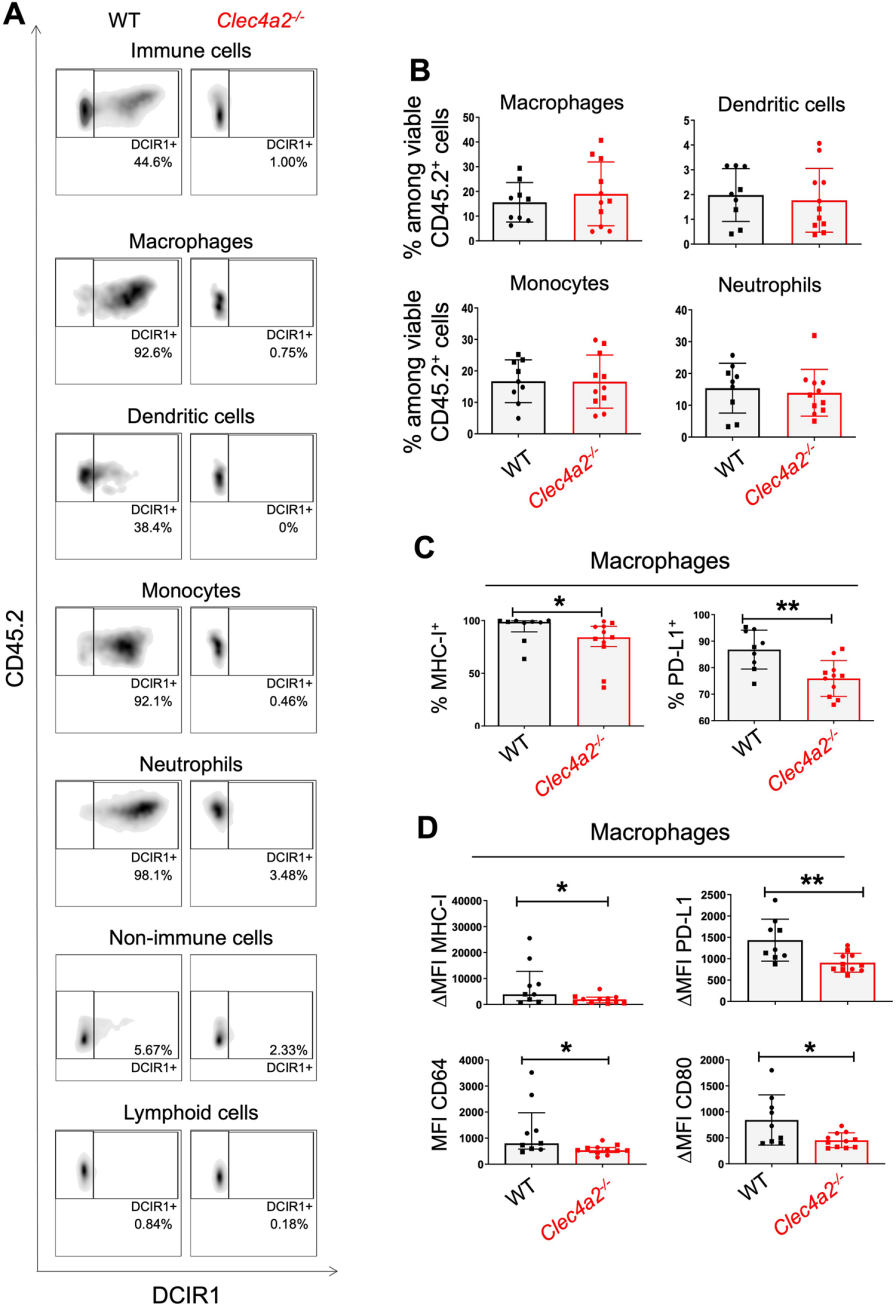
DCIR1 deficiency modulates innate immune response during CRC development in mouse. FACS analysis of tumor-infiltrating myeloid cells in WT and DCIR1 deficient (*Clec4a2*^*−/−*^) mice at day 29 after IC injection of MC38-fLuc^+^ cells. (**A**) Representative FACS plots of DCIR1 expression at the cell surface of tumor-infiltrating myeloid cell subsets, non-immune cells (CD45.2^−^) and lymphoid cells (CD45.2^+^ CD11b^−^ CD11c^−^) from WT and *Clec4a2*^*−/−*^ mice. (**B**) Percentage of tumor-infiltrating myeloid cell subsets. (**C**) Percentage of MHC-I^+^ or PD-L1^+^ tumor-associated macrophages. (**D**) Median fluorescence intensity (MFI) or differential median fluorescence intensity (ΔMFI) of MHC-I, PD-L1, CD64 and CD80 at the cell surface of tumor-associated macrophages. Panels B to D were generated from two independent experiments (n = 9 WT and n = 11 *Clec4a2*^*−/−*^ mice). Different symbols (i.e., dots and squares) are used to indicate independent experiments. *P < 0.05, **P < 0.01.

As both human DCIR and mouse DCIR1 are mainly expressed by myeloid cells, we next characterized the phenotype of these cells in CRC mouse tumors using flow cytometry. As expected, DCIR1 was detected on tumor-infiltrating monocytes, macrophages, dendritic cells and neutrophils but not on stromal/cancer cells (CD45.2^−^) and lymphoid cells (CD45.2^+^ CD11b^−^ CD11c^−^) (Fig. 4A and supplementary Fig. S3A,B; Gating strategy in supplementary Fig. S4A). Absence of DCIR1 in mice did not modify the percentage of the different tumor-infiltrating myeloid cell populations, as measured at day 29 after MC38-fLuc^+^ cells implantation (Fig. 4B). However, analysis of the activation markers (CD64, PD-L1), co-stimulatory molecules (CD80) and antigen-presenting molecules (MHC-I and MHC-II) on myeloid cells revealed profound differences between tumor-bearing WT and *Clec4a2*^*−/−*^ mice. First, we noticed that the percentage of MHC-I^+^ and PD-L1^+^ tumor-associated macrophages (TAMs) was significantly lower in *Clec4a2*^*−/−*^ tumors compared to WT tumors (Fig. 4C). The percentage of tumor-infiltrating MHC-I^+^, but not PD-L1^+^, dendritic cells, monocytes, and neutrophils was also significantly lower in *Clec4a2*^*−/−*^ mice (Supplementary Fig. S3C). Second, we found a lower median fluorescence intensity (MFI) of MHC-I, PD-L1, CD64 and CD80 in TAMs from *Clec4a2*^*−/−*^ mice compared to their WT counterparts (Figs. 4D). *Clec4a2*^*−/−*^ TAMs also trended to exhibit lower MFI of MHC-II (Supplementary Fig. S3D). In contrast to TAMs, other tumor-infiltrating myeloid cell populations showed comparable levels of these different markers in WT and *Clec4a2*^*−/−*^ mice, with the exception of *Clec4a2*^*−/−*^ neutrophils for which we observed a decrease in MFI of MHC-II (Supplementary Fig. S3E). We next analyzed myeloid cell subsets in the colon-draining mesenteric lymph nodes (mLNs) where adaptive immune cells are primed by antigen-presenting cells (Gating strategy in supplementary Fig. S4B). However, we observed no difference in the percentage of myeloid cell subsets nor in their expression of CD64, CD80, PD-L1, MHC-I and MHC-II (Supplementary Fig. S5). Taken together, these results suggest that DCIR1 deficiency alters the activation status of myeloid cells, especially TAMs, in CRC tumors.

Type I and II interferons (IFNs) are strong inducers of the expression of MHC class I, class II, PD-L1 and co-stimulatory molecules (CD80, CD86, CD40) on both dendritic cells and macrophages, hence boosting dendritic cells antigen cross-presentation and macrophages pro-inflammatory phenotype^42,43^. As we previously showed that dendritic cells from *Clec4a2*^*−/−*^ mice presented an impaired response to type I and II IFNs^17^, we wondered if this could explain the phenotypic alterations observed in *Clec4a2*^*−/−*^ TAMs. To test this hypothesis, we used flow cytometry to measure the expression of MHC-I, MHC-II and CD86 in WT and *Clec4a2*^*−/−*^ bone-marrow derived macrophages (BMDMs) and dendritic cells (BMDCs) with or without stimulation with increasing concentrations of type I (IFN-α and IFN-β) or type II IFNs (IFN-γ). Although IFN stimulations increased the expression of these proteins at the cell surface of BMDMs and BMDCs, we observed no difference between WT and *Clec4a2*^*−/−*^ cells (Supplementary Fig. S6A). In line with these results, our qPCR data revealed no significant differences in the gene expression of several IFN-γ and type I IFN responsive genes (*Nos2*, *Stat1*, *Cxcl9*, *Cxcl10*, *Cxcl11*, *Cx3cl1*) between WT and *Clec4a2*^*−/−*^ tumors (Supplementary Fig. S6B). Another plausible explanation for the altered phenotype of *Clec4a2*^*−/−*^ TAMs in CRC is that DCIR1 may play a crucial role in tissue adaptation of macrophages, as recently demonstrated for vascular macrophages^22^. Therefore, we analyzed the content of myeloid cells and their cell surface markers in the colon of resting WT and *Clec4a2*^*−/−*^ mice (Supplementary Fig. S7). However, we found no difference between WT and *Clec4a2*^*−/−*^ mice. We thus concluded that while the phenotypic differences observed in *Clec4a2*^*−/−*^ tumor-infiltrating myeloid cells are driven by tumor development, they are likely not attributable to an alteration in their IFN response.

### DCIR1 deficiency impairs the adaptive immune response during CRC in mice

**Figure 5:**
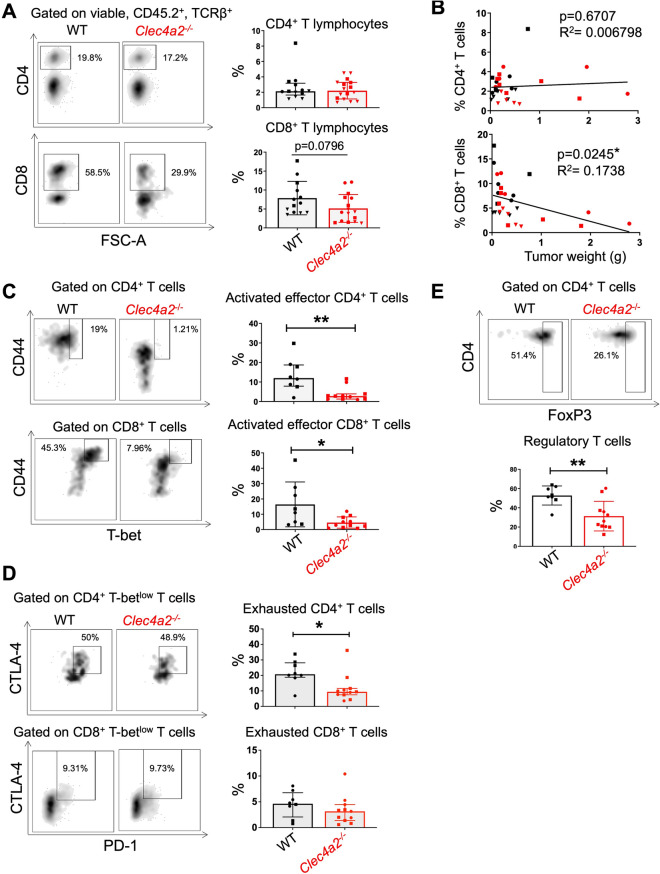
DCIR1 deficiency alters the adaptive immune response during CRC development in mouse. FACS analysis of tumor-infiltrating T lymphocytes in WT and DCIR1 deficient (*Clec4a2*^*−/−*^) mice at day 29 after IC injection of MC38-fLuc^+^ cells. (**A**) Representative FACS plots (left panels) and percentage (right panels) of tumor infiltrating CD4^+^ and CD8^+^ T cells (n = 13 WT and n = 16 *Clec4a2*^*−/−*^ mice). (**B**) Correlation between tumor weight and the percentage of tumor-infiltrating CD4^+^ or CD8^+^ T cells. (**C**,**D**) Representative FACS plots and percentage of tumor infiltrating (**C**) activated effector (**D**) exhausted CD4^+^ and CD8^+^ T lymphocytes (n = 8 WT and n = 11 *Clec4a2*^*−/−*^ mice) and (**E**) regulatory T cells. *P < 0.05, **P < 0.01.

The myeloid immune compartment orchestrates adaptive immune cells^44^ and using our syngeneic orthotopic CRC mouse model, we have previously demonstrated that these cells, particularly CD8^+^ T cells lymphocytes, act as key effectors cells restricting MC38 tumor growth^39^. We therefore investigated whether the altered myeloid phenotype observed in *Clec4a2*^*−/−*^ mice could lead to an alteration of the adaptive immune response, ultimately resulting in increased tumor growth. Using flow cytometry, we analyzed tumor-infiltrating T lymphocytes in both WT and *Clec4a2*^*−/−*^ mice at day 29, post-tumor implantation. We found no difference in CD4^+^ T cell infiltration between WT and *Clec4a2*^*−/−*^ tumors (Fig. 5A; Gating strategy in supplementary Fig. S8) and no association between the percentage of intra-tumor CD4^+^ T cells and tumor weight (Fig. 5B). Regarding the infiltration of CD8^+^ T cells, we observed that lower proportions of CD8^+^ T lymphocytes trended to infiltrate tumors from *Clec4a2*^*−/−*^ mice as compared with WT mouse tumors (Fig. 5A). In line with the major antitumor effector functions of CD8^+^ T lymphocytes, their intra-tumor infiltration negatively correlated with tumor weight in both WT and *Clec4a2*^*−/−*^ animals (Fig. 5B). Activated effector T helper 1 CD4^+^ (Th1) and Type 1 CD8^+^ cells (Tc1) strongly express both the cell surface protein CD44 and the intracellular transcription factor T-bet^45–48^. We found that the percentage of Th1 (CD4^+^, CD44^Hi^ T-bet^Hi^) and Tc1 cells (CD8^+^, CD44^Hi^ T-bet^Hi^) was significantly lower in *Clec4a2*^*−/−*^ tumors than in WT tumors (Fig. 5C). The decrease of Th1 and Tc1 lymphocytes observed in *Clec4a2*^*−/−*^ tumors did not lead to an increase in Foxp3^+^ cells accounting regulatory T cells (Treg) as a reduction in the percentage of Treg was observed in DCIR1 deficient mice compared to WT animals (Fig. 5E). When CD8^+^ and CD4^+^ T lymphocytes are chronically activated, such as in the context of cancer, their effector functions are progressively inhibited, particularly through the expression of immune checkpoint receptors, including PD-1 and cytotoxic T-lymphocyte-associated protein 4 (CTLA-4)^49^. Yet, the diminished frequency of Th1 and Tc1 cells observed in *Clec4a2*^*−/−*^ tumors could not be attributed to increased cell exhaustion since we did not observe a higher percentage of T-bet^low^ CTLA-4^+^ PD-1^+^ CD4^+^ and CD8^+^ T cells in *Clec4a2*^*−/−*^ tumors compared with WT (Fig. 5D). It should be noted that a significant proportion of the exhausted T-bet^low^ CTLA-4^+^ PD-1^+^ CD4^+^ T cells were Foxp3^+^ Treg cells (around 70% and 50% in tumors of WT and *Clec4a2*^*−/−*^ mice), the remaining part most likely being truly exhausted CD4^+^ T cells (Supplementary Fig. S9A). Finally, given the well-described role of NK cells, γδ T lymphocytes and Th17 cells in regulating CRC growth^49,50^, we analyzed their infiltration in WT and *Clec4a2*^*−/−*^ tumors but found no difference between mouse lines (Supplementary Fig. S9B; Gating strategy in supplementary Fig. S8).

We next analyzed the T cell compartment in the colon-draining mesenteric lymph nodes (mLNs) where T lymphocytes are primed by antigen-presenting cells. While no difference was observed in the frequency of CD4^+^ T cells, CD8^+^ T cells, Treg and Th1 cells (CD4^+^, CD44^Hi^ T-bet^+^) in mLNs of WT and *Clec4a2*^*−/−*^ tumor-bearing mice, we found a lower percentage of Tc1 cells (CD8^+^, CD44^Hi^ T-bet^+^) in the mLNs of *Clec4a2*^*−/−*^ mice (Supplementary Fig. S10A; Gating strategy in supplementary Fig. S10B). Based on these results, we concluded that during CRC development in mice, DCIR1 deficiency impairs the activation of CD4^+^ and CD8^+^ T cells in tumors, and the activation of CD8^+^ T cells in the mLNs.

## Discussion

The present study describes that DCIR contributes to the immune response and pathogenesis of colorectal cancer.

First, we observed that high DCIR gene (*CLEC4A*) expression is associated with an improved survival of CRC patients, as others have reported^26,30^. Second, we found positive correlations between *CLEC4A* expression and the presence of different innate immune cell subtypes in the CRC tumor, which is not surprising at first glance considering that DCIR is mainly expressed by myeloid cells. More remarkable, however, is the positive correlations between *CLEC4A* expression and the abundance of distinct T cell subsets as well as high immunologic constant of rejection, reflecting a Th1/cytotoxic-oriented immune response. Collectively, these findings suggest a regulatory role of DCIR in anti-tumor immunity. Of note, correlations between *CLEC4A* expression and tumor-infiltration of several innate and adaptive immune cells were also reported in (cutaneous) melanoma and hepatocarcinoma^27–29^, suggesting that DCIR regulates the immune response in multiple types of cancer.

In agreement with human transcriptomic CRC data, we demonstrated that DCIR1 deficiency altered the adaptive immune response and promotes tumor growth in our orthotopic pre-clinical CRC mouse model. Since adaptive immunity is essential for mediating tumor rejection and for controlling tumor growth in our model^39^, the reduced percentage of activated effector CD4^+^ and CD8^+^ T cells observed in tumors of *Clec4a2*^*−/−*^ mice likely accounts for the increased tumor growth observed in these mice. In contrast, a recent study showed a reduction in AOM/DSS-induced colitis associated tumorigenesis in *Clec4a2*^*−/−*^ mice compared with WT mice as a consequence of decreased inflammation in *Clec4a2*^*−/−*^ mice in response to DSS^24^. Thus, DCIR1 would play a detrimental role in an inflammation-induced colorectal tumorigenesis (AOM/DSS) model but a protective role when cancer cells have settled and tumor development is controlled by adaptive immunity, as mimicked by our orthotopic mouse CRC model.

In line with our data, other studies showed that *Clec4a2*^*−/−*^ mice are characterized by a defect in T cell recruitment and/or activation in various inflammatory contexts^18–21^. For instance, Maglinao and coworkers found that *Clec4a2*^*−/−*^ mice present a decreased activation of splenic CD4^+^ and CD8^+^ T cells and a lower infiltration of T cells in the brain during experimental cerebral malaria^19^. However, we and others have previously established that, in autoimmune and infectious settings, *Clec4a2*^*−/−*^ mice develop an exacerbated adaptive immune response, notably reflected by an increase in IFN-γ-producing CD4^+^ T cells^14–17,23^. The regulatory function of DCIR/DCIR1 in immunity must therefore most certainly depend on the tissue and pathological context. Consistent with this idea, our data showed that DCIR1 deficiency promotes the growth of CRC tumors in mice but has no impact on the development of subcutaneous tumors, even when we injected the same cancer cell line (i.e., MC38-Fluc^+^). It is unlikely that the microbiome accounts for this difference because i/ recombinant DCIR only binds to a marginal portion of commensal gut microbes^51^ and ii/ DCIR-regulated immune response was found to be independent of gut microbiota in (DSS)-induced colitis and colon cancer^24^. Furthermore, we have previously shown that tumor rejection versus progression in mice is associated with differences in the very early colonic immune response, most likely shaped by the microbiota^39^. Nonetheless, we observed no difference in the the percentages of tumor-progressing versus tumor-rejecting mice were similar in WT and *Clec4a2*^*−/−*^ animals. Instead, we proposed that this difference is related to tissue-specific immune responses. Accordingly, a recent study reported significant differences in the immune response when MC38 cells are injected subcutaneously or into the colon^52^, fueling the growing body of research suggesting that the tumor microenvironment differs considerably between organs^53,54^. Specifically, a higher level of tumor-infiltrating T cell and NK cells (around 3 times more) was measured in orthotopic CRC tumors compared to subcutaneous tumors^52^. As a consequence, orthotopic CRC showed a greater sensitivity to immune checkpoint blockade compared to subcutaneous tumors, demonstrating the important role of T cells in mediating tumor rejection within colon. Thus, DCIR1 may play a more prominent role in regulating tumor development within the colon rather than subcutaneously, due to the greater magnitude of the adaptive immune response and its importance in restricting tumor growth within the colon.

As compared to WT mice, DCIR1-deficient mouse showed reduced activation of CD4^+^ and CD8^+^ T cells in tumors, and activation of CD8^+^ T cells in the mLNs. The reduced percentage of activated CD4^+^ and CD8^+^ T cells observed in tumors from *Clec4a2*^*−/−*^ mice is probably not due to a defect in T cell recruitment, since we observed no clear difference in the total number of CD4^+^ and CD8^+^ T cells or in the expression of chemokines (*Cxcl9-11* and *Cx3cl1*) attracting these cells. Our data suggest that the defective adaptive immune response in *Clec4a2*^*−/−*^ mice is related to altered myeloid cell functions, in particular those related to antigen presentation and T cell activation. TAMs represent the most abundant myeloid cells in tumors. It is generally accepted that TAMs promote tumor progression and metastasis, and TAM infiltration is associated with poor prognostic outcome and reduced overall survival in multiple human tumor types^55^. However, these associations are not as clear in colorectal cancer. In fact, there is considerable heterogeneity of TAMs in human CRC, and some studies have shown that infiltration of immature monocyte-derived TAMs or TAM subsets correlates with improved overall survival in patients with CRC^56–58^. Certain TAM subsets may therefore play an antitumor role in CRC. We showed that DCIR1 is expressed on tumor-infiltrating myeloid cells but not on T lymphocytes, and that DCIR1 deficiency was associated with a reduced cell surface expression of molecules for antigen presentation in TAMs (notably MHC-I, CD80 and to a lesser extent MHC-II). Although macrophages are in general much less efficient than DCs at presenting antigens to T cells, some studies have demonstrated that intestinal macrophages might be involved in antigen presentation to previously activated T cells locally^59–61^. Notably, intestinal macrophages can capture and transfer antigens to CD103^+^ dendritic cells, by a route involving gap junctions and MHC, thereby playing an essential role in promoting adaptive immunity^60^. Future work will focus on determining whether colonic TAMs contribute directly or indirectly to antigen presentation in our CRC mouse model and whether the altered phenotype of *Clec4a2*^*−/−*^ TAMs explains the reduced adaptive immune response. Besides TAMs, we also noticed a decrease in MHC-I expression levels in monocytes and DCs in colon tumors from *Clec4a2*^*−/−*^ mice compared to WT mice. Like DCs, colon-specific monocyte-derived macrophage precursors have been shown, under inflammatory conditions, to display migratory and antigen-presenting capacities that allow them to prime naive T cells^62^. The decrease in MHC-I expression in tumor-infiltrating monocyte-derived cells and DCs of *Clec4a2*^*−/−*^ mice could therefore also explain the decreased adaptive immune response, particularly Tc1, observed in those mice.

The reasons why DCIR1 deficiency alters the phenotype of CRC-infiltrating myeloid cells, particularly TAMs, remain elusive. Interestingly, a recent publication demonstrated that macrophages and dendritic cells, but not T lymphocytes, express not only DCIR but also its ligand(s)^23^. This suggests that DCIR can interact in cis with its ligand(s) to regulate intrinsic myeloid cell functions. Accordingly, we previously showed that DCs from *Clec4a2*^*−/−*^ mice presented an impaired response to type I IFN as compared to WT cells^17^. We hypothesized that this could explain the phenotype of *Clec4a2*^*−/−*^ TAMs but we failed to recapitulate this phenotype after stimulation of bone marrow-derived macrophages or dendritic cells with various concentrations of type I and II IFNs. We also examined the possibility that DCIR1 may play a crucial role in the tissue adaptation of these cells, as recently demonstrated for vascular macrophages^22^. Again, we did not find differences in the colon-specific myeloid cell compartment when comparing WT and *Clec4a2*^*−/−*^ mice in the basal state. The only conclusion that can be drawn from these data is that the phenotype observed in the *Clec4a2*^*−/−*^ myeloid cells is linked to tumor development.

In summary, by revealing a hitherto unknown role for DCIR in the complex picture of immunity to cancer, our study paves the way for DCIR exploitation in the context of CRC treatment and beyond. Yet, further work is needed to clarify the role played by DCIR in antitumor immunity and in unravelling the mechanisms underlying the phenotypic changes observed in the myeloid and T-cell compartments in tumors from *Clec4a2*^*−/−*^ mice. In particular, it would be interesting to monitor the innate and adaptive immune response during colorectal cancer progression. Furthermore, given the broad but still unclear impact of DCIR in immunity, we need to gain a solid understanding of the functional role of DCIR in each cell subtype expressing it, by generating conditional knockout mice, and to characterize the still unknown ligand(s) of this receptor.

### Supplementary Information

Supplementary Information.

## Data Availability

The data that supports the findings of this study are available from the corresponding author upon reasonable request.
