# Supplementary material for: The C-type lectin DCIR contributes to the immune response and pathogenesis of colorectal cancer
Source: Sci Rep. 2024 Mar 26;14:7199. doi: 10.1038/s41598-024-57941-y (PMC10966077; doi:10.1038/s41598-024-57941-y)
Supplement: Supplementary file 1 — Supplementary Information. [file 41598_2024_57941_MOESM1_ESM.docx]

**SUPPLEMENTAL MATERIALS**

#### The C-type lectin DCIR contributes to the immune response and pathogenesis of colorectal cancer

**Authors and affiliations:** Giulia Trimaglio^1^, Tamara Sneperger^1#^, Benjamin B. A. Raymond^1#^, Nelly Gilles^1^, Emmanuelle Näser^1^, Marie Locard-Paulet ^1^, Marieke E. Ijsselsteijn^2^, Thomas P. Brouwer^2^, Romain Ecalard^3^, Jessica Roelands^2^, Naoki Matsumoto^4^, André Colom^1^, Myriam Habch^1^, Noel F.C.C. de Miranda^2^, Nathalie Vergnolle^5^, Christel Devaud^5^, Olivier Neyrolles^1^, Yoann Rombouts^1^*

^1^ Institut de Pharmacologie et de Biologie Structurale (IPBS), Université de Toulouse, CNRS, UPS, Toulouse, France.

^2^ Department of Pathology, Leiden University Medical Center, Leiden, The Netherlands.

^3^ Zootechnie PURPAN INSERM/UPS/ENVT US 006 CREFRE, Pavillon Lefebvre - CHU Purpan, 31024, Toulouse, France.

^4^ Department of Integrated Biosciences, Graduate School of Frontier Sciences, The University of Tokyo, 5-1-5 Kashiwanoha, Kashiwa, Chiba, 277-8562, Japan.

^5^ Institut de Recherche en Santé Digestive (IRSD), Université de Toulouse, INSERM, INRAe, ENVT, UPS, Toulouse, France.

^#^ These authors contributed equally and are ordered randomly

* Correspondence: [yoann.rombouts@ipbs.fr](file:///C:\Users\giu_t\AppData\Local\Temp\yoann.rombouts@ipbs.fr)

**Contents**

Supplementary Table 1. List of the antibodies used in flow cytometry
Supplementary Table 2. List of the primers used in RT-qPCR
Supplementary Figures 1-10 and legends

Supplementary Table 1

| **Reactivity** | **Conjugate** | **Company** | **Clone** | **Dilution** | **Staining conditions** |
| --- | --- | --- | --- | --- | --- |
| B220 | eFluor450 | eBioscience | RA3 6B2 | 1:200 | Extracellular |
| CD11b | BV785 | Biolegend | M1/70 | 1:200 | Extracellular |
| CD11b | BV650 | Biolegend | M1/70 | 1:200 | Extracellular |
| CD11c | PE | BD Biosciences | HL3 | 1:100 | Extracellular |
| CD11c | PE-Cy7 | Biolegend | N418 | 1:200 | Extracellular |
| CD4 | BV510 | Biolegend | RM4-5 | 1:200 | Extracellular |
| CD4 | BV786 | BD Biosciences | RM4-5 | 1:200 | Extracellular |
| CD44 | BV650 | Biolegend | IM7 | 1:200 | Extracellular |
| CD45.2 | BUV737 | BD Biosciences | 104 | 1:100 | Extracellular |
| CD64 | Pe-Dazzle 594 | Biolegend | X54-5/7.1 | 1:100 | Extracellular |
| CD80 | BV650 | Biolegend | 16-10A1 | 1:100 | Extracellular |
| CD80 | PE-Dazzle | Biolegend | 16-10A1 | 1:100 | Extracellular |
| CD86 | PE | eBioscience | GL1 | 1:200 | Extracellular |
| CD8a | FITC | Biolegend | 53-6.7 | 1:200 | Extracellular |
| CD8a | BV650 | Biolegend | 53-6.7 | 1:200 | Extracellular |
| CTLA4 | BV605 | Biolegend | UC10-4B9 | 1:100 | Intracellular |
| CD16/32 | - | Biolegend | 93 | 1:100 | Extracellular |
| FoxP3 | AF700 | Thermo Fisher Scientific | FJK-16s | 1:100 | Intracellular |
| mDCIR1 | AF647 | Gift from Naoki Matsumoto | TKKT1 | 1:500 | Extracellular |
| Ly6C | eFluor 450 | Thermo Fisher Scientific | HK1.4 | 1:100 | Extracellular |
| Ly6G | AF700 | BD Pharmigen | 1A8 | 1:100 | Extracellular |
| Viability | - | Thermofisher scientific | - | 1:500 | Extracellular |
| MHC-I | Pe/Cy7 | Biolegend | AF6-88.5 | 1:800 | Extracellular |
| MHC-I/H-2Kb | FITC | Biolegend | AF6-88.5 | 1:200 | Extracellular |
| MHC-II | AF488 | Biolegend | M5/114.15.2 | 1:400 | Extracellular |
| MHC-II | APC-Cy7 | Biolegend | M5/114.15.2 | 1:200 | Extracellular |
| NK-1.1 | BV510 | Biolegend | PK136 | 1 :200 | Extracellular |
| PD1 | BV711 | BD OptiBuild | J43 | 1:200 | Extracellular |
| PD-L1 | BV711 | Biolegend | 10F.9G2 | 1:200 | Extracellular |
| RORgt | PE | BD Pharmingen | Q31-378 | 1 :100 | Intracellular |
| T-bet | PeCy7 | Thermo Fisher Scientific | 4B10 | 1:100 | Intracellular |
| TCRbeta | AF700 | Biolegend | H57-597 | 1:200 | Extracellular |
| TCRbeta | APC-Cy7 | Biolegend | H57-597 | 1:200 | Extracellular |
| TCRgamma/delta | BV421 | BD Biosciences | B1 | 1:200 | Extracellular |

Supplementary Table 2

|  |  |  |  |
| --- | --- | --- | --- |
| Gene | Primers | | Company |
| *Cx3cl1* | Fwd (5'-3') | CAGTGGCTTTGCTCATCCGCTA | Sigma |
|  | Rev (5'-3') | AGCCTGGTGATCCAGATGCTTC |  |
| *Cxcl10* | Fwd (5'-3') | ATTGCCACGATGAAAAAGAATGAT |  |
|  | Rev (5'-3') | AGACCCAAGGGCAATTAGGACTAGC |  |
| *Cxcl11* | Fwd (5'-3') | CCGAGTAACGGCTGCGACAAAG |  |
|  | Rev (5'-3') | CCTGCATTATGAGGCGAGCTTG |  |
| *Cxcl9* | Fwd (5'-3') | AGCAGTGTGGAGTTCGAGGAAC |  |
|  | Rev (5'-3') | AGGGATTTGTAGTGGATCGTGC |  |
| *iNOS* | Fwd (5'-3') | TCCTCACGCTTGGGTCTTGTTC | Sigma aldrich (Merck) |
|  | Rev (5'-3') | TCCAACGTTCTCCGTTCTCTTGC |  |
| *Stat1* | QT00162183 | | QIAGEN |

**Supplementary figure 1: *CLEC4A* expression and its association with patient overall survival according to the stages of colon cancer.**

(**A**) Spearman correlation (Rs= -0.24, P = 3.85e-7) between *CLEC4A* expression in primary tumor samples of the TCGA-COAD dataset (n=439) and pathologic tumor stages as defined by American Joint Committee on Cancer (AJCC). NA: Not available. (**B**) Association between *CLEC4A* expression and overall survival (OS) of patients stratified by different stages of colon cancer (TCGA-COAD dataset). Cox proportional hazard regression analysis was performed to calculate HR and corresponding P value. Error bars indicate the 95% confidence interval of the HR. HR: hazard ratio.

**Supplementary figure 2: DCIR1 deficiency has no impact on subcutaneous tumor development in mice.**

**A-C**: Tumor size measurements from WT and DCIR1 deficient (*Clec4a2*^-/-^) mice injected subcutaneously with (**A**) B16F1 (1 experiment, n=8 mice for each group), (**B**) B16F10 (1 experiment, n=10 mice for each group until day 10 while n=9 mice for each group on day 13) or (**C**) MC38-fLuc^+^ cell lines (2 independent experiments pooled, n=16 WT and n=17 *Clec4a2*^-/-^ mice).

**Supplementary Figure 3: Characterization of tumor-infiltrating myeloid cells in WT and *Clec4a2^-/-^* mice.**

FACS analysis of tumor-infiltrating myeloid cells in WT and *Clec4a2^-/-^* mice at day 29 after IC injection of MC38-fLuc^+^ cells. **A**: Percentage of DCIR1^+^ cells among tumor-infiltrating myeloid cells at day 29 after IC injection of MC38-fLuc^+^ cells, as measured by FACS (n=9 WT mice). **B**: Differential median fluorescence intensity (ΔMFI) of DCIR1 at the cell surface of tumor-infiltrating myeloid cells at day 29 after IC injection of MC38-fLuc^+^ cells (n=5 WT mice; one of two experiments). **C:** Percentage of MHC-I^+^ tumor-infiltrating dendritic cells, monocytes and neutrophils. **D:** ΔMFI of MHC-II at the cell surface of tumor-associated macrophages. **E**: MFI or ΔMFI of CD64, CD80, PD-L1, MHC-I and MHC-II at the cell surface of tumor-infiltrating dendritic cells, monocytes and neutrophils. Panels C to E were generated from two independent experiments (n=9 WT mice and n=11 *Clec4a2*-KO). Different symbols (*i.e.,* dots and squares) are used to indicate independent experiments (2 independent experiments pooled). *P< 0.05.

**Supplementary Figure 4: Gating strategy of myeloid cells in the tumors and tumor-draining mesenteric lymph nodes.**

FACS analysis of myeloid cells in the tumors and mLNs of mice at day 29 after IC injection of MC38-fLuc^+^ cells. **A**: Gating strategy (derived from representative staining of one WT mouse) showing non-immune cells (gated as live, CD45.2^-^ cells), neutrophils (gated as live, CD45.2^+^, Ly6G^+^, CD11b^+^ cells), monocytes (gated as live, CD45.2^+^, Ly6C^+^, CD11b^+^ cells after exclusion of neutrophils), macrophages (gated as live, CD45.2^+^, CD11b^+^, CD64^+^ cells after exclusion of neutrophils and monocytes), dendritic cells (gated as live, CD45.2^+^, MHC-II^+^, CD11c^+^ cells after exclusion of neutrophils, monocytes and macrophages) and lymphoid cells (gated as CD45.2^+^ viable, CD11b^-^, CD11c^-^ cells) from the tumors analyzed at day 29 after tumor implantation. **B**: Gating strategy for macrophages (gated as CD45.2^+^ viable, CD11b^+^, CD64^+^ cells), conventional dendritic cells (gated as CD45.2^+^ viable, B220^-^, CD11c^+^, MHC-II^+^ cells after exclusion of macrophages) and plasmacytoid dendritic cells (gated as CD45.2^+^ viable, B220^+^, CD11c^+^, MHC-II^+^ after exclusion of macrophages) from the mLNs of tumor-bearing mice at day 29 after tumor implantation.

**Supplementary Figure 5: DCIR1 deficiency does not affect the phenotype of myeloid cells in tumor-draining mesenteric lymph nodes of mice.**

FACS analysis of myeloid cells in the tumor-draining mLNs of WT and *Clec4a2^-/-^* mice at day 29 after IC injection of MC38-fLuc^+^ cells. **A:** Percentages of macrophages, conventional dendritic cells and plasmacytoid dendritic cell. **B**: Median fluorescence intensity (MFI) or differential median fluorescence intensity (ΔMFI) of CD64, CD80, PD-L1, MHC-I, MHC-II in macrophages, conventional dendritic cells and plasmacytoid dendritic cells. Each symbol corresponds to a single mouse. Different symbols (*i.e.,* dots and squares) are used to indicate independent experiments (2 independent experiments pooled).

**Supplementary Figure 6: BMDMs and BMDCs from WT and *Clec4a2*-KO mice are equally responsive to IFNs.**

**A**: Percentages and MFI of MHC-I^+^, MHC-II^+^ or CD86^+^ bone marrow-derived macrophages and dendritic cells stimulated or not for 24 hours with increasing concentrations of IFN-α (**left panels**), IFN-β (**center panels**) and IFN-γ (**right panels**). Representative experiment (out of 2 independent experiments) in which simulations and analyses were performed in duplicate. **B**: Expression of IFN-responsive genes (*Nos2*, *Stat1*, *Cxcl9-11*, *Cx3cl1*), as measured by real-time qPCR, in tumors from WT and *Clec4a2*^-/-^ mice at day 29 after IC injection of MC38-fLuc^+^ cells. Each symbol corresponds to a single mouse (2 independent experiments pooled, n=6-8 WT and n=6-7 *Clec4a2*^-/-^ mice). ns: non-significant.

**Supplementary Figure 7: DCIR1 deficiency does not affect the phenotype of myeloid cells in the colon of resting mice.** FACS analysis of myeloid cells, gated as described in supplementary Figure 4, in the colon of resting WT and *Clec4a2*^-/-^ mice. **A**: Percentage of macrophages, dendritic cells, monocytes and neutrophils. **B**: Percentage and MFI of CD64^+^, CD80^+^, PD-L1^+^, MHC-I^+^ and MHC-II^+^ macrophages, dendritic cells, monocytes and neutrophils. Each symbol corresponds to a single mouse (n=5 WT and n=4 *Clec4a2*^-/-^ mice).

**Supplementary Figure 8: Gating strategy of T cells in the tumors.**

FACS analysis of T lymphocytes in the tumors of WT and *Clec4a2^-/-^* mice at day 29 after IC injection of MC38-fLuc^+^ cells. Gating strategy (derived from representative staining of one WT mouse) showing γδ^+^ T lymphocytes (gated as CD45.2+ viable, TCRγδ^+^ cells), NK cells (gated as CD45.2+ viable, NK1.1^+^ cells), CD8^+^ T cells (gated as CD45.2^+^ viable, TCRβ^+^, CD8^+^ cells), CD4^+^ T cells (gated as CD45.2^+^ viable, TCRβ^+^, CD4^+^ cells), regulatory T cells (gated as FoxP3^+^ among CD4^+^ T cells), Th17 cells (gated as RORgt^+^ among CD4^+^ T cells), activated effector CD8^+^ T cells (gated as T-bet^Hi^, CD44^Hi^ among CD8^+^ T cells), exhausted CD8^+^ T cells (gated as T-bet^Low^, CTLA-4^+^, PD-1^+^ among CD8^+^ T cells), activated effector CD4^+^ T cells (gated as T-bet^Hi^, CD44^Hi^ among CD4^+^ T cells) and exhausted CD4^+^ T cells (gated as T-bet^Low^, CTLA-4^+^, PD-1^+^ among CD4^+^ T cells).

**Supplementary Figure 9: Tumor-infiltrating immune cells.**

**A**: Percentage of Foxp3^+^ and Foxp3^-^ among the exhausted T-bet^low^ CTLA-4^+^ PD-1^+^ CD4^+^ T cells. **B**: Percentage of tumor-infiltrating γδ+ T lymphocytes, Th17 lymphocytes and NK cells (as gated in supplementary Figure 8) in the tumors of WT and *Clec4a2^-/-^* mice at day 29 after IC injection of MC38-fLuc^+^ cells. Different symbols (*i.e.,* dots and squares) are used to indicate independent experiments (n=8 WT and n=11 *Clec4a2^-/-^* mice).

**Supplementary Figure 10: Gating strategy of T cells in the tumor-draining mesenteric lymph nodes.**

FACS analysis of T lymphocytes in the tumor-draining mLNs of WT and *Clec4a2^-/-^* mice at day 29 after IC injection of MC38-fLuc^+^ cells. **(A)** Percentage of total and activated effector CD4^+^ and CD8^+^ T cells and total regulatory T cells. **(B)** Gating strategy (derived from representative staining of one WT mouse) showing CD8^+^ T cells (gated as CD45.2^+^ viable, TCRβ^+^, CD8^+^ cells), CD4^+^ T cells (gated as CD45.2^+^ viable, TCRβ^+^, CD4^+^ cells), regulatory T cells (gated as FoxP3+ among CD4^+^ T cells), activated effector CD8^+^ T cells (gated as CD44^Hi^, T-bet^+^ among CD8^+^ T cells) and activated effector CD4^+^ T cells (gated as CD44^Hi^, T-bet^+^ cells among CD4^+^ T cells).
